# Synchrony as a measure of conversation difficulty: Movement coherence increases with background noise level and complexity in dyads and triads

**DOI:** 10.1371/journal.pone.0258247

**Published:** 2021-10-05

**Authors:** Lauren V. Hadley, Jamie A. Ward

**Affiliations:** 1 Hearing Sciences—Scottish Section, School of Medicine, University of Nottingham, Glasgow, United Kingdom; 2 Computing, Goldsmiths, University of London, London, United Kingdom; University of Connecticut, UNITED STATES

## Abstract

When people interact, they fall into synchrony. This synchrony has been demonstrated in a range of contexts, from walking or playing music together to holding a conversation, and has been linked to prosocial outcomes such as development of rapport and efficiency of cooperation. While the basis of synchrony remains unclear, several studies have found synchrony to increase when an interaction is made challenging, potentially providing a means of facilitating interaction. Here we focus on head movement during free conversation. As verbal information is obscured when conversing over background noise, we investigate whether synchrony is greater in high vs low levels of noise, as well as addressing the effect of background noise complexity. Participants held a series of conversations with unfamiliar interlocutors while seated in a lab, and the background noise level changed every 15-30s between 54, 60, 66, 72, and 78 dB. We report measures of head movement synchrony recorded via high-resolution motion tracking at the extreme noise levels (i.e., 54 vs 78 dB) in dyads (n = 15) and triads (n = 11). In both the dyads and the triads, we report increased movement coherence in high compared to low level speech-shaped noise. Furthermore, in triads we compare behaviour in speech-shaped noise vs multi-talker babble, and find greater movement coherence in the more complex babble condition. Key synchrony differences fall in the 0.2–0.5 Hz frequency bands, and are discussed in terms of their correspondence to talkers’ average utterance durations. Additional synchrony differences occur at higher frequencies in the triads only (i.e., >5 Hz), which may relate to synchrony of backchannel cues (as multiple individuals were listening and responding to the same talker). Not only do these studies replicate prior work indicating interlocutors’ increased reliance on behavioural synchrony as task difficulty increases, but they demonstrate these effects using multiple difficulty manipulations and across different sized interaction groups.

## 1. Introduction

When people hold conversations, they take turns talking with the goal of exchanging information. Much prior work on behaviours relevant to conversation has explored the production and comprehension processes in turn, often measuring individuals talking into a microphone or listening to prerecorded speech in isolation. However, assuming that the behaviour of an isolated individual extrapolate to a conversation situation is problematic as it ignores the interdependence of behaviours [1], and interdependent behaviours have the potential to reveal new insights into social interaction [2]. Furthermore, conversation does not only comprise speech production and comprehension; in daily life conversation most often occurs face to face, giving interlocutors the ability to additionally produce and comprehend nonverbal signals. Nonverbal signals include visual, tactile, and proprioceptive cues, which have been shown to vary according to both the internal and external context of the conversation [3,4].

Here we address the nonverbal behaviours of people conversing in a multi-person face-to-face conversation, specifically focusing on interpersonal synchrony: how people align their movement behaviours in time. There is a long history of research into synchrony in conversation, dating back to the study of cyclicity vs synchronicity in the 1980s [5–7]. Synchrony has been measured using equipment ranging from video cameras during natural conversation to, more recently, motion tracking systems during lab-based studies; can be analysed using correlation, recurrence, or spectral methods [8]; and has been demonstrated in conversation behaviours ranging from speech rate to gesture [9]. Importantly when studying synchrony, pseudo interactions, in which participants are shuffled into fake groupings, can be generated to measure a baseline level of synchrony regardless of method [10–12]. To date, research has shown a wide variety of high- and low-level contextual factors to affect synchrony during conversation, from type of conversation to concurrent perceptual signals [13–15].

Increased synchrony has been linked to a variety of benefits, including mutual comprehension and feelings of rapport [16–18]. In fact, recent work indicates that the synchrony of participants’ head movements can be used to predict success of information exchange in a conversation [19], and behavioural synchrony has been proposed to be a core mechanism of successful interaction [20]. There are several theories regarding how synchrony leads to these beneficial effects. One possibility is that synchrony reflects the means by which an individual represents their partner in relation to themselves. The common coding theory proposes that perception and production of an action are dependent on the same cognitive structures [21]. In other words, to understand the actions that an individual observes another person producing, they engage the mechanisms that they use to produce that action themselves: i.e., having a common code for perception and production. This proposition is supported by evidence of embodied cognition, whereby one’s own bodily experience affects how one perceives the actions of another [22], and the finding that one’s own motor system is activated when perceiving an action similarly to when producing that action [23]. A related theory, of prediction-by-simulation, takes this proposition a step further to propose that it is through this use of one’s own motor system during perception that individuals are able to predict what their interaction partner is likely to do next [24,25]. Importantly, a number of prior studies have found that engagement of one’s motor system while observing a partner is enhanced if understanding the partner’s action is challenging. For example, enhanced motor activation has been shown when listening to speech in a noisy compared to quiet background [26], or when listening to speech produced in a distorted manner [27], suggesting a compensatory role which could facilitate interaction success. If engagement of the motor system and interpersonal synchrony are based on the same mechanism, it is likely that more challenging interaction situations would also lead to greater behavioural synchrony [28].

Several studies have found behavioural synchrony to be enhanced during more difficult interactive tasks, with a variety of different manipulations of difficulty leading to higher levels of synchrony. For example, a study by Wallot and colleagues used a joint model-building paradigm [29], manipulating interaction structure as a means of adjusting difficulty (i.e., allowing dyads to work on Lego cars jointly, placing one in a director role, or requiring strict turn taking–difficulty order confirmed via participants’ subjective evaluation). The more difficult the task, the greater the (wrist movement) synchrony between individuals. In another study, Louwerse and colleagues used a map description conversation paradigm, in which an instruction giver describes a path to a listener who has an equivalent map missing the path [30]. By obscuring the listener’s map in ways that made description easier or harder, the effect of communication difficulty on behavioural synchrony was analysed. When communication was more challenging, interpersonal synchrony of both head movement and gesture increased. Finally, an exploratory study by Boker and colleagues explored the effect of a noisy vs quiet background on movement synchrony in four conversing dyads [15]. They again found greater difficulty to relate to greater synchrony, in terms of both head and hand movement.

Here we specifically focus on behaviour while people converse in noise. Many of our conversations in everyday life are in noisy environments, from the café to the train station, and these environments can make communication particularly challenging. Louder levels of noise lead to a variety of compensatory nonverbal behaviours, from adjustments of speech parameters to greater use of gesture [31,32]. However, it is not only the level of noise that affects nonverbal behaviour; the type of noise is also important. Both the frequency and the temporal spectra of the background noise impacts behavioural adjustments [33]. However, use of speech as a background noise is a special case, as background speech competes not only with the spectrum of target speech, but with its linguistic content, further increasing communicative difficulty [34]. In background speech, target speech intelligibility is reduced [35], and several potentially facilitatory nonverbal behaviours are enhanced compared to noise without linguistic information [36,37].

In this paper we investigate whether movement synchrony increases in line with communication difficulty during free conversation. We measure synchrony using cross wavelet coherence, a time-frequency spectrum method that is well suited to uncovering coordination between two or more timeseries [38,39]. Coherence has successfully been used to study dyadic synchrony during music performance [40], joke telling [41], and conversation [11,12], and allows different forms of coordinated behaviour to be investigated through analysis of different frequency ranges. Importantly, different frequency ranges can be related to different forms of movement. For example, movement relating to breathing is reflected in frequencies of around 0.2–0.3 Hz [42], whereas movement relating to rapid postural shifts is reflected in frequencies of over 2.2 Hz [43]. Therefore, coherence at these different frequency ranges could indicate synchrony of breathing rate, or postural shifts, respectively.

We build on previous work finding increased use of simulation in challenging communication situations, and increased behavioural synchrony in difficult interaction tasks, to address the use of synchrony to facilitate conversation in noisy conversations of both dyads and triads. We specifically focus on overall motion energy of the head, to include behaviours ranging from adjustments of head orientation and partner proximity, to use of nods or shakes. We manipulate the difficulty of communication by varying the level of background noise, and the type of background noise. In terms of noise level, we compare the extremes of 54 dB noise (i.e., quiet, lower than typical vocal levels in conversation [44]), and 78 dB noise (i.e., loud, close to the maximum typically reported in restaurants [45]). In terms of the type of background noise, we compare speech-shaped noise (SSN) with eight-talker babble as they have similar frequency spectra, but only babble provides informational masking [34]. We predict that interlocutors will be more synchronised when talking in a high than a low noise level. Furthermore, we predict that interlocutors will be more synchronised when talking against more complex babble noise than the simpler speech-shaped noise.

## 2. Method

### 2.1. Participants

Thirty unacquainted native Glaswegian participants were organised into mixed gender dyads (age M  =  61 years, SD  =  11 y), and thirty-three unacquainted native Glaswegian participants were organised into mixed-gender triads (age M = 61 y, SD = 11 y). This study was approved by the West of Scotland Research Ethics Committee (09/S0704/12), and participants were paid £10 each for taking part. Written consent was obtained from all participants.

### 2.2. Materials and task

**Fig 1:**
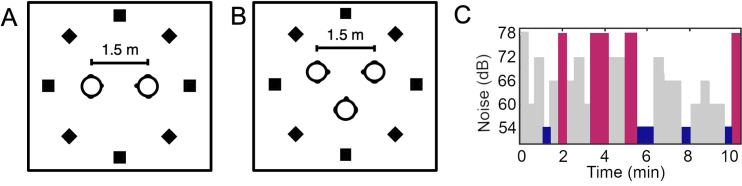
Study setup. (A) shows the positions of loudspeakers and participant chairs for dyads, and (B) shows positions for triads. (C) shows an example of the noise level changes as a function of time (varying between 54, 60, 66, 72, and 78 dB).

Full details of setup have been previously published [4,37]. To summarise, participants in both the dyad and triad experiments were seated in a sound attenuated room within a ring of eight equidistantly spaced loudspeakers (diameter 3.6 m). Participants were seated 1.5m from each other (one in front of the other in the dyad experiment, and in an equilateral triangle in the triad experiment). See Fig 1. A Vicon motion tracking system was used to record head movement coordinates (in relation to the centre of the room), and movement was sampled at 100 Hz. Eye movement and speech recording were also collected, but are not reported here.

Participants conversed while background noise was presented continuously from the loudspeakers at 54, 60, 66, 72, or 78 dB. Noise level changed every 15-30s according to a paired de Bruijn sequence with 10ms of smoothing between segments to avoid startle responses. In other words, the background changed regularly between the five noise levels in a way that was unpredictable for participants. Each conversation lasted for approximately 10 minutes in total. For the dyad experiment, the noise presented was uncorrelated speech-shaped noise, based on the long-term-average spectrum generated from the speech data of Byrne and colleagues [46]. For the triad experiment, two noise types were used: uncorrelated speech-shaped noise as above, and eight-talker babble produced by concatenating sentences (four males, from [47]). The experiment was controlled by Matlab and a bespoke Max/MSP patch.

### 2.3. Procedure

Participants were introduced and taken into the booth. They were then fit with motion tracking crowns, microphones, and eye trackers (taking approx. 40 minutes). Any participants that wore hearing aids were asked to remove them for the experiment. Dyads held three conversations in speech-shaped noise; triads help two conversations in speech-shaped noise and two conversations in eight-talker babble (order of noise types randomised between triads). The conversation topics for dyads focused on films [48], close-call incidents [49], and an ethical dilemma [50]. The conversation topics for triads additionally included a discussion of Glasgow history. The order of conversation topics was counterbalanced (between dyads, and between triads), and the experimenter entered the booth to give participants the starter for each conversation between trials. Participants were told that noise would be playing and would sometimes be loud, but were encouraged to continue conversing regardless.

### 2.4. Analysis

We use cross wavelet coherence analysis to evaluate the amount of head movement synchrony within dyads (and within the constituent pairs of triads). Typically used to analyse long-term timeseries in geophysics [38], wavelet coherence has recently been used to study conversational synchrony in dyads [11,12] and in triads [51]. Wavelet coherence reveals the amount of time and frequency-domain correlation between two signals, regardless of the power in the different frequency components. This means that synchrony is not dependent on the energy of the movement, and thus that synchronous micro-movements can elicit coherence values as large as synchronous posture changes. One advantage of the method being based on wavelets, as opposed to the commonly used Fourier transform, is that it works well with non-stationary data (it does not assume perfectly rhythmic movements) and thus can better capture the changing dynamics of free conversation [39].

Cross wavelet coherence is calculated by first applying a wavelet transform to each of the signals being compared, combining these to create the cross-wavelet transform (by multiplication of one transform with the complex conjugate of the other), and finally converting this into a coherence spectrogram [39]. The spectrogram charts coherence at different sized frequency bands (corresponding to wavelet scales) across time, with values ranging from 0 (uncorrelated) to 1 (correlated). By averaging the spectrogram over a set time period, a response can be obtained for that time period which charts the frequencies at which the two signals are coordinated [12]. That is, if two people tend to move together at periods of say, 2 s, the analysis will reveal a higher average coherence at 0.5 Hz (i.e., the frequency corresponding to movements occurring every 2 s).

The motion capture data is first pre-processed by extracting the x, y, and z coordinates at the centre of each participant’s head (identified through referencing the motion tracking crown to a pair of removable motion tracking goggles indicating the ears and bridge of the nose). These data are low-pass filtered using an 8^th^ order Butterworth filter with cutoff 30 Hz, and are standardised across each full conversation recording. The 3-axes are then combined using Euclidean norm to create a single signal representing the total head movement of each participant. We then divide the data into segments according to each noise condition, and calculate a cross wavelet coherence spectrogram for each.

Wavelet coherence is calculated using the Matlab toolbox provided by [38], with the default parameters (Morlet wavelet, w = 6). We additionally specify 36 wavelet periods ranging from approximately 0.13 to 7.7 s. To mitigate the effect of edge conditions between adjacent segments, we remove the so-called cone of influence [38]. The average coherence across time is finally calculated over all segments from each condition to produce a single frequency response for each dyad.

In the triadic case, the response from each of the three constituent pairwise analyses are averaged to produce a single response for each triad. Wavelet coherence is well defined as a pairwise measure of synchrony, however it is less well defined how it might be adapted for three or more signals. An algorithm based on multiple wavelet coherence [52] was previously used on triadic conversations [51]. However, that approach produces different results depending on which signal is chosen as the ‘dependent’ signal [52]. Our approach avoids this issue by addressing pairwise behaviour within the triad and averaging those pairwise responses.

We present two forms of analysis within each group size and noise type. First, we analysed coherence in real vs pseudo conversations at each noise level. Pseudo conversations were created conservatively, by reshuffling recorded behaviour of participants from the same dyad/triads measured in the same background noise level. For example, for dyad AB, the behaviour measured from participant A recorded in the first segment of 54 dB background noise was aligned with the behaviour of participant B recorded in the second segment of 54 dB. Segments that were unequal in duration were cut to the shortest segment’s duration. Second, we removed pseudo conversation coherence from real conversation coherence to get a corrected level of synchrony that we compared between noise levels. Removing the pseudo conversation coherence meant that only synchrony within the group that was specific to the dynamics of the real conversation was retained. Finally, we present one analysis in the triads to compare behaviour in SSN and babble: a comparison of corrected coherence at each noise level to identify the effect of noise complexity. Note that these datasets have previously been analysed in terms of how individual speaking and listening behaviours change in different levels and types of background noise [4,37], but dynamic behaviours between interlocutors have not been addressed. Given our new focus on whether interpersonal synchrony indexes communication difficulty, this repurposing of data is not considered dual publication.

## 3. Results

All comparative results are calculated using paired t-tests on N = 14 dyads (1 dyad from the original 15 was discarded due to artefacts), or N = 11 triads. Significance is tested at p<0.05 with an additional 0.05 False Detection Rate (FDR) adjustment for multiple comparisons [53]. Effect size is measured using Cohen’s-d [54]. This is done for each of 36 different wavelet scales, which cover synchronous periods of between 0.13 s and 7.7 s (the lower limit being based on prior work on quick conversational movements [12], and the upper limit being based on the duration of half of the minimum segment length). Note that if only a single frequency band is significant, we consider it an outlier and do not discuss it further.

### 3.1. Dyads: Effects of noise level

**Fig 2:**
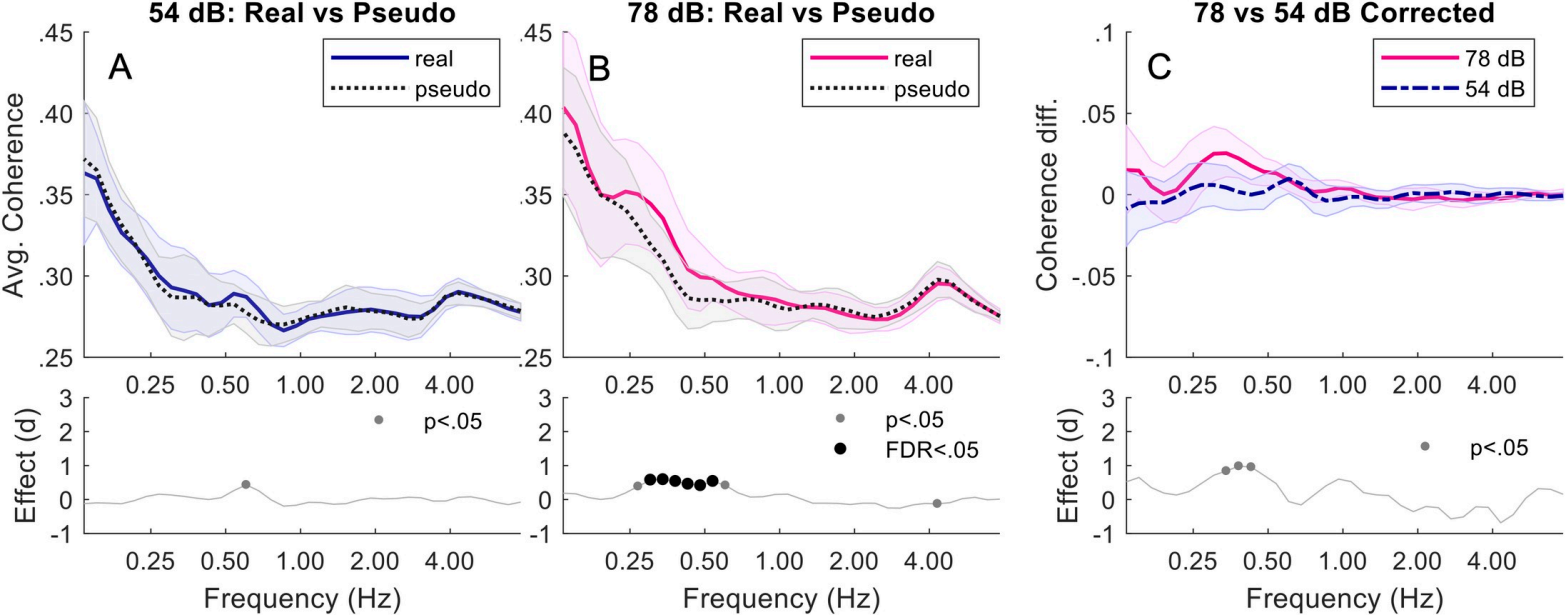
Dyads in speech-shaped noise. Top plots of (A) and (B) show the average wavelet coherence responses for Real vs Pseudo during 54 dB and 78 dB respectively. The mean coherence over all dyads (with standard error) is shown across a range of frequencies. The lower plots show the corresponding Pseudo-Real effect size (Cohen’s d), with both significance and FDR-adjusted significance (if any). (C) shows the corrected (baseline removed) coherence difference between 78 and 54 dB.

Comparing pseudo and real dyads in SSN, at 78 dB, real dyads were more synchronised than pseudo dyads between 0.27–0.61 Hz (*p*s < .04). Only 0.34–0.54 Hz (*p*s < .006) remained significant after FDR adjustment. At 54 dB, there was no significant difference between real and pseudo dyads. Corrected coherence was greater in 78 dB noise than 54 dB noise between 0.34–0.43 Hz (*p*s < .05). When FDR adjustment was applied this was not significant. See Fig 2.

### 3.2. Triads: Effects of noise level and complexity

**Fig 3:**
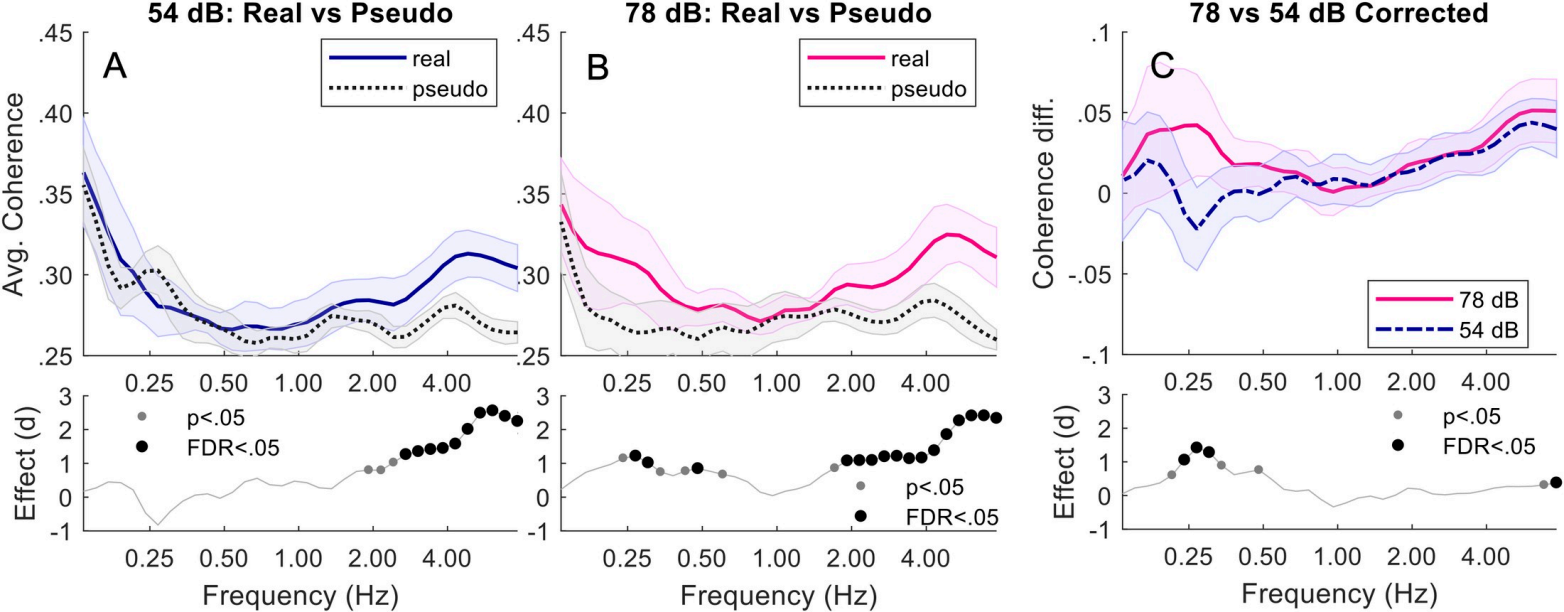
Triads in speech-shaped noise. Top plots of (A) and (B) show the average wavelet coherence responses for Real vs Pseudo during 54 dB and 78 dB respectively. The mean coherence over all triads (with standard error) is shown across a range of frequencies. The lower plots show the corresponding Pseudo-Real effect size (Cohen’s d), with both significance and FDR-adjusted significance. (C) shows the corrected (baseline removed) coherence difference between 78 and 54 dB.

Speech-Shaped Noise: Comparing pseudo and real triads, at 78 dB, real triads were more synchronised than pseudo triads between 0.24–0.61 Hz (*p*s < .05). Only 0.27–0.3 and 0.48 Hz (*p*s < .02) remained significant after FDR adjustment. Real triads were also more synchronised than pseudo triads at 78 dB at higher frequencies between 1.7–7.4 Hz (*p*s < .03). The band 1.9–7.42 Hz (*p*s < .02) remained significant after FDR adjustment. At 54 dB, real triads were only more synchronised than pseudo triads at the higher frequencies of between 1.9–7.3 Hz (*p*s < .04). The band 2.7–7.4 Hz (*p*s < .003) remained significant after FDR adjustment. Corrected coherence was greater in 78 dB noise than 54 dB noise between 0.21–0.48 Hz (*p*s < .04). Only 0.24–0.3 Hz (*p*s < .002) remained significant after FDR adjustment. Corrected coherence was also greater in 78 dB noise than 54 dB noise at higher frequencies between 0.68–7.4 Hz (*p*s < .01). Only 7.4 Hz (*p* = .004) remained significant after FDR adjustment. See Fig 3.

**Fig 4:**
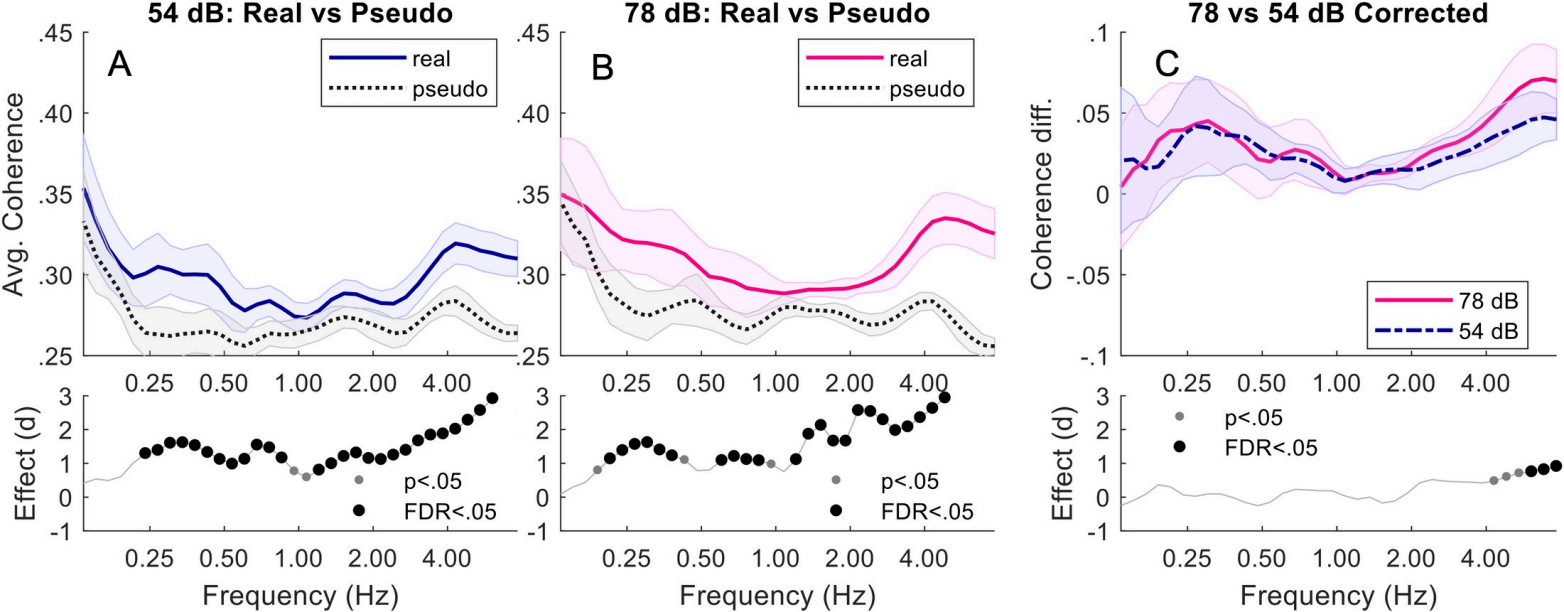
Triads in babble. Top plots of (A) and (B) show the average wavelet coherence responses for Real vs Pseudo during 54 dB and 78 dB respectively. The mean coherence over all triads (with standard error) is shown across a range of frequencies. The lower plots show the corresponding Pseudo-Real effect size (Cohen’s d), with both significance and FDR-adjusted significance. (C) shows the corrected (baseline removed) coherence difference between 78 and 54 dB.

Babble: Comparing pseudo and real triads, at both 54 dB and 78 dB, real triads were significantly more synchronised than pseudo triads across almost the entire analysed frequency spectrum. Corrected coherence was only greater in 78 dB noise than 54 dB noise at the higher frequencies of between 4.3–7.4 Hz (*p*s < .04). The band 5.7–7.4 Hz (*p*s < .005) remained significant after FDR adjustment. See Fig 4.

**Fig 5:**
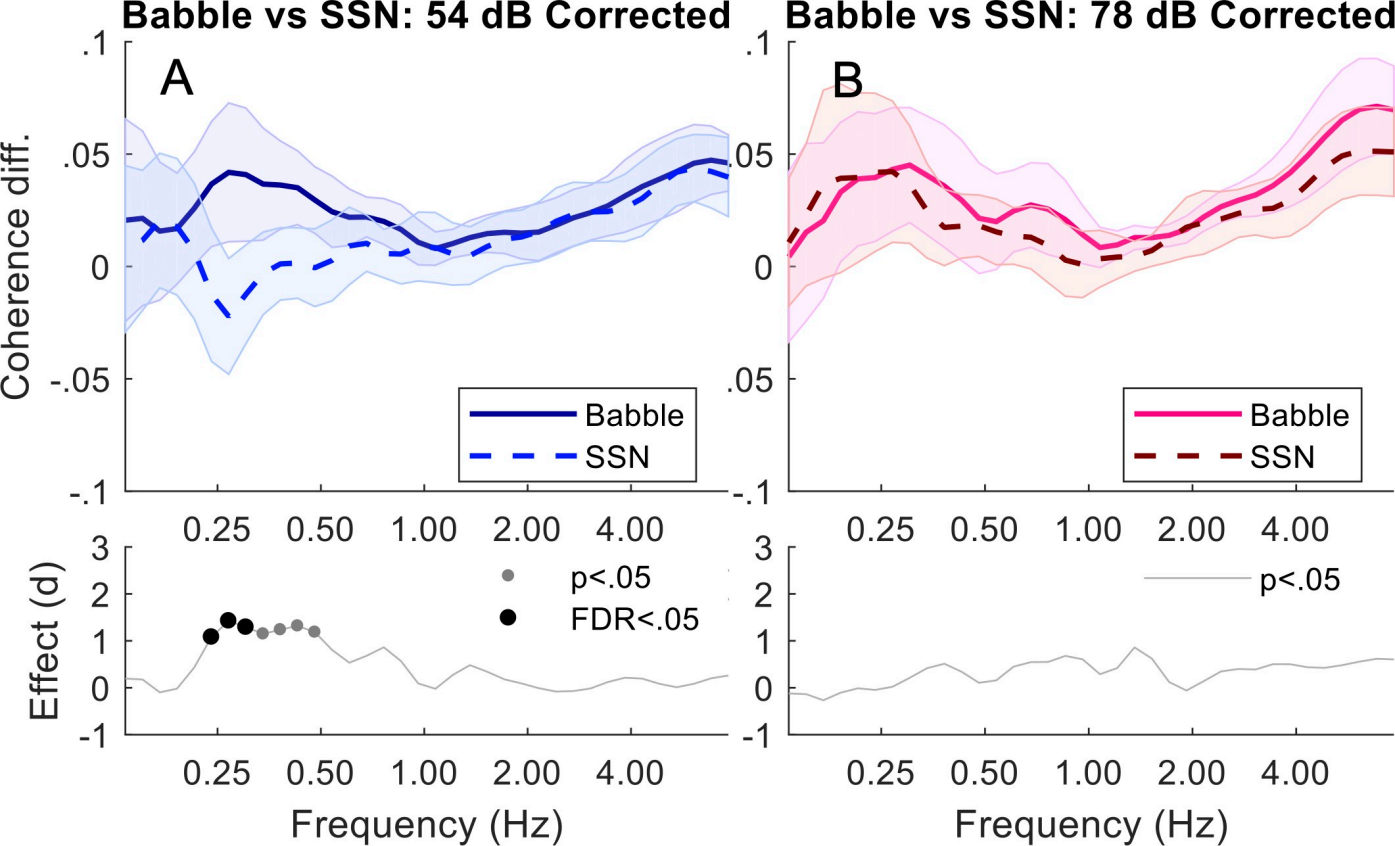
Triads in speech-shaped noise vs babble. Top plots of (A) and (B) show corrected (baseline removed) coherence responses comparing SSN vs Babble conditions for 54 and 78 dB. Cohen’s d effect sizes, with corresponding significance points (if any), are shown in the lower plots.

Comparing Speech-Shaped Noise and Babble: At 54 dB, corrected coherence was greater in babble than speech shaped noise between 0.24–0.48 Hz (*p*s < .02). Only 0.24–0.3 Hz (*p*s < .002) remained significant after FDR adjustment. At 78 DB, there was no difference between babble and speech-shaped noise. See Fig 5.

## 4. Discussion

In this paper, we analysed the movement coherence of dyads and triads conversing in different levels and types of background noise. In dyads and triads conversing over speech-shaped noise, we found noise level effects on synchrony at lower frequencies. Furthermore, in triads, we found noise level effects on synchrony at higher frequencies, as well as an effect of noise complexity. In all cases, we found greater synchrony in more difficult communication conditions, as hypothesised. This is a novel investigation of synchrony in conversational groups of different sizes, and is one of relatively small number of studies of triadic groups [51,55]. We consider the detail of these effects in turn below.

First, we focus on low-frequency coherence. For conversation over speech-shaped noise, synchrony in real dyads was significantly greater than in pseudo dyads at 78 dB, but not at 54 dB, an effect that we replicated in triads. Furthermore, comparing corrected coherence at different noise levels revealed a trend in dyads, and a significant effect in triads, for greater synchrony in higher levels of noise. For conversation over more complex babble noise, synchrony in real triads was significantly greater than in pseudo triads at both noise levels, and corrected coherence was greater in the more complex babble noise than the speech-shaped noise at 54 dB. In sum, we found evidence for greater synchrony with increased background noise in both dyads and triads when conversing over simple speech-shaped noise, and for greater synchrony in more complex noise in triads. Remarkably, the frequencies at which these effects occur correspond well to the average duration of utterances in the different size groups. In dyads, average utterance duration was 2.74s [4], and we saw a trend for differences in corrected coherence at 0.34–0.43 Hz, corresponding to periods of 2.3–2.9 s. In triads, average utterance duration was 3.71 s [37], and we saw significant differences in corrected coherence at 0.24–0.3 Hz, corresponding to periods of 3.3–4.2 s. We therefore suggest that the differences in head movement synchrony that we report may relate to turn-taking, providing a means of smoothing the interaction as difficulty communicating increases.

Turning to high-frequency coherence, synchrony in real triads was significantly greater than synchrony in pseudo triads in all types and levels of noise. Furthermore, corrected coherence was greater in high noise levels than low noise levels in both the speech-shaped and babble noise (with no difference being found between noise types). Interestingly, no effects were evident in high frequencies in dyads. While we are not able to further probe the basis of this very quick movement behaviour, we suggest that it could relate to the high-frequency backchannelling behaviour reported by Hale and colleagues [12]. They found listeners to show quick nodding behaviour during the speech of their partner. As the structure of a triadic conversation necessitates there being two listeners at any one time, it is likely that they are both simultaneously producing backchannel behaviour. The greater high frequency synchrony in real than pseudo triads could therefore be the result of the pair of listeners indirectly synchronising while responding to the same talker–something that would not occur in dyads as only one person would be backchannelling at a time. Since backchannelling has been proposed to provide positive evidence of mutual understanding [56], it may be particularly salient when communication difficulty is high. While we cannot test this explanation (due to interlocutors’ short speech turns leading to very short segments for analysis), this would be a valuable question for future work, employing a paradigm developed to elicit longer conversational turns.

Interpersonal synchrony is clearly ubiquitous, but the mechanism by which synchrony occurs between individuals remains unclear. It is of course difficult to infer from movement data alone the mechanism and function of synchrony. While we have shown that the frequency of synchronous movements relates to the duration of utterances, our data do not allow us to address how or why these behaviours relate. Further insight could be generated by triangulating different datastreams from the conversation, such as body movement, details of the conversation structure, and linguistic content. For example, are interlocutors more synchronised when they alternate quickly between speaking and listening turns, when they agree on a topic, or when their language also aligns? Comprehensive study of the interrelation of conversation signals are critical to begin to understand the function of synchrony in conversational interaction.

Nonetheless, our findings, of increased synchrony in difficult communication conditions, provide additional support to prior suggestions that synchrony is the result of an action-perception link [57]. A range of work has demonstrated that activation of a listener’s motor system when listening to speech increases when the intelligibility of that speech is reduced [26,58]. Such motor activity could therefore act to facilitate comprehension, and ease interaction [24,25]. We suggest that synchrony with a conversation partner may therefore be an outward manifestation of the internal motor simulation of that partner’s speech, explaining the greater levels of synchrony in the more challenging communication conditions. This could be tested by simultaneously investigating the neural activity and movement synchrony of interlocutors, to assess whether increases in motor activity are coupled with measurements of interpersonal synchrony.

However, while we have reported novel synchrony effects that both generalise across dyads and triads, and relate to other behavioural measures (i.e., utterance duration), we recognise that this work has several limitations. First, the study included frequent changes in background noise, with segments of each level lasting 15-30s. This does not reflect a typical listening experience, and also led to some very short segments following data cleaning (i.e., removing the cone of influence). Such a design therefore means that synchrony can only be confidently analysed down to approximately 0.13 Hz, which could obscure effects at longer timescales. Second, as participants were seated it is likely that this setup led movement to be relatively constrained; there may have been greater movement and movement synchrony had they been standing. Finally, we also note that we had a relatively small sample size, and used a highly conservative measure of pseudo coherence, which may have obscured some effects. In detail, to determine pseudo coherence, we used the two/three participants that were in a real grouping, but took different segments of the same conversation and artificially overlaid them. Hence if consistent movement patterns emerged between interactors over the course of a conversation, these will also have been present in the pseudo conversation and thus will have been removed when calculating corrected coherence. Lack of power, and conservative baselining, may therefore have contributed to the effects of noise level not surviving in the corrected coherence dyad analysis.

This paper presented an analysis of the head movement of dyads and triads holding conversations in different levels and types of noise. We hypothesised that higher noise levels, and more complex noise types, would lead to greater coherence between interlocutors due to the greater communicative difficulty. We found supporting evidence in both dyads and triads. When conversing over speech-shaped noise, we found a trend for dyads, and a significant effect for triads, to be more synchronised at higher noise levels. Furthermore, when comparing triads conversing over speech-shaped and babble noise, we found them to be more synchronised in the complex babble noise than the simple speech-shaped noise. These findings complement prior work finding greater evidence of motor engagement in challenging noise environments [26], and are consistent with simulation being engaged by interlocutors to facilitate conversational turn-taking.
